# High rate of autonomic neuropathy in Cornelia de Lange Syndrome

**DOI:** 10.1186/s13023-021-02082-y

**Published:** 2021-10-30

**Authors:** M. J. Pablo, P. Pamplona, M. Haddad, I. Benavente, A. Latorre-Pellicer, M. Arnedo, L. Trujillano, G. Bueno-Lozano, L. M. Kerr, S. A. Huisman, F. J. Kaiser, F. Ramos, A. D. Kline, J. Pie, B. Puisac

**Affiliations:** 1 Unit of Clinical Genetics and Functional Genomics, Department of Pharmacology-Physiology, School of Medicine, University of Zaragoza, CIBERER-GCV02 and IIS-Aragon, Zaragoza, Spain; 2 Unit of Neurophysiology, San Jorge University Hospital, Huesca, Spain; 3 Unit of Neurophysiology, Miguel Servet University Hospital, Zaragoza, Spain; 4 Unit of Clinical Genetics, Department of Pediatrics, Hospital Clinico Universitario “Lozano Blesa”, CIBERER-GCV02 and IIS-Aragon, Zaragoza, Spain; 5 Department of Pediatrics, Hospital Clinico Universitario “Lozano Blesa”, Growth, Exercise, Nutrition and Development (GENUD) Research Group, Zaragoza, Spain; 6 Division of Pediatric Neurology, Department of Paediatrics, University of Utah Health, Salt Lake City, UT USA; 7 Department of Pediatrics, Amsterdam UMC, Amsterdam, The Netherlands; 8 Prinsenstichting, Purmerend, The Netherlands; 9 Institute of Human Genetics, University Hospital Essen University of Duisburg-Essen, Essen, Germany; 10 Harvey Institute of Human Genetics, Greater Baltimore Medical Center, Baltimore, MD USA

**Keywords:** Cornelia de Lange Syndrome, CdLS, Small fiber nerve, Peripheral neuropathy, Autonomic neuropathy, Sudomotor test, Sweat gland density, NIPBL gene

## Abstract

**Background:**

Cornelia de Lange Syndrome (CdLS) is a rare congenital disorder characterized by typical facial features, growth failure, limb abnormalities, and gastroesophageal dysfunction that may be caused by mutations in several genes that disrupt gene regulation early in development. Symptoms in individuals with CdLS suggest that the peripheral nervous system (PNS) is involved, yet there is little direct evidence.

**Method:**

Somatic nervous system was evaluated by conventional motor and sensory nerve conduction studies and autonomic nervous system by heart rate variability, sympathetic skin response and sudomotor testing. CdLS Clinical Score and genetic studies were also obtained.

**Results:**

Sympathetic skin response and sudomotor test were pathological in 35% and 34% of the individuals with CdLS, respectively. Nevertheless, normal values in large fiber nerve function studies.

**Conclusions:**

Autonomic nervous system (ANS) dysfunction is found in many individuals with Cornelia de Lange Syndrome, and could be related to premature aging.

**Supplementary Information:**

The online version contains supplementary material available at 10.1186/s13023-021-02082-y.

## Background

Cornelia de Lange Syndrome (CdLS) is a genetic disease due to spontaneous mutations in genes of the cohesin protein complex, mainly *NIPBL*, in 70% of the cases [1–4] and *SMC1A*, *SMC3*, *RAD21*, *BRD4*, *HDAC8, ANKRD11* and *MAU2* [5–9]. Manifestations of the syndrome differ with mutated gene type, with variants in *NIPBL* often associated with more severe clinical phenotype. The syndrome is characterized by typical facial features, growth failure, limb abnormalities and the involvement of many organs and systems including the central nervous system. Sweating abnormalities, abnormal reactions to cold and heat, and severe gastrointestinal reflux are also prevalent and suggest a compromised peripheral nervous system [1]. More than 80% of individuals with CdLS have some autonomic nervous system dysfunction, while 26% of those have moderate to severe dysfunction as measured by the Compass-31 questionnaire, a validated survey tool for autonomic dysfunction [10]. The aim of this study was to get new insights into neuronal dysfunction in CdLS by analyzing large and small fiber nerves with different techniques.

## Patients and methods

All the peripheral nervous system studies, except the sudomotor test, were made in a group of 20 individuals with CdLS (7 male, 13 female, aged 3–37 years). In the sudomotor test the population was broadened to 47 individuals with CdLS (18 male, 29 female, aged 1.5–42 years) and 50 slightly older healthy controls (18 male, 32 female, aged 7–48 years). All of the individuals with CdLS and controls were Caucasian, except 3 Latino and 1 Middle East subjects in the CdLS group. The protocol study was approved by the Ethics Committee of Clinical Research from the Government of Aragón (CEICA;PI16/225). All the individuals with CdLS and controls gave informed consent for their participation.

To evaluate the somatic peripheral nervous system, conventional motor and sensory nerve conduction studies [11–15] were carried out in upper and lower limbs (large fiber nerves).

The autonomic nervous system (small fibre nerves) was studied by means of heart rate variability at rest, sympathetic skin response and sudomotor test. Heart rate variability (HRV) at rest was evaluated recording the heart rate for 5 min [16]. Sympathetic skin response (SSR) was studied with electric stimuli over the Median and Posterior Tibial nerves, recording the responses over the palm of both hands (Median) and the sole of both feet (Tibial) [17, 18]. Nerve conduction studies, HRV and SSR were performed by the same group of neurophysiologists with a 5-channel Natus® Electromyography equipment. The sudomotor test, which gives the number of functioning sweat glands per cm^2^ (sweat gland density, SGD) was obtained on a silicone mold after pilocarpine iontophoresis stimulation over the foot dorsum [19].

Genetic studies were realized by standard Sanger sequencing and Next Generation Sequencing (NGS) panels. Clinical severity score according to the first international consensus statement [1] was also studied (Table 1). Statistical studies were achieved with the SPSS program version 25.

**Table 1:** CdLS clinical score (severity)

Individuals with CdLS	1	2	3	4	5	6	7	8	9	10	11	12	13	14	15	16	17	18	19	20	21	22	23	24
**Cardinal features (2 points each if present)**																								
Synophrys and/or thick eyebrows	+	+	+	+	+	+	+	+	–	+	+	–	–	+	+	+	+	+	–	+	+	+	+	+
Short nose, concave nasal ridge and/or upturned nasal tip	+	–	+	+	+	+	+	–	–	+	+	–	+	+	–	+	–	–	–	+	+	–	+	–
Long and/or smooth philtrum	+	–	–	+	+	+	+	–	–	+	+	+	–	+	+	–	+	–	–	+	+	+	+	–
Thin upper lip vermilion and/or downturned corners of mouth	+	+	–	+	+	+	+	–	–	+	+	+	–	+	+	–	+	–	–	+	+	+	+	–
Hand oligodactyly and/or adactyly	–	–	–	–	–	–	–	–	–	–	–	–	–	–	–	–	–	–	–	+	+	–	–	–
Congenital diaphragmatic hernia	–	–	–	–	–	+	–	–	–	–	–	–	–	–	–	–	–	–	–	–	–	–	–	–
**Suggestive features (1 point each if present)**																								
Global developmental delay and/or intellectual disability	+	+	+	+	+	+	+	+	+	+	+	+	+	+	+	–	+	+	+	+	+	+	+	+
Prenatal growth retardation (< 2 SD)	+	–	+	+	+	+	+	–	+	–	+	–	–	+	–	–	+	–	+	+	+	+	+	+
Postnatal growth retardation (< 2 SD)	+	–	+	+	+	+	+	+	+	–	–	+	–	+	–	+	+	–	+	+	+	+	+	+
Microcephaly (prenatally and/or postnatally)	–	–	+	+	+	+	+	+	+	+	+	–	–	+	–	–	+	+	–	+	+	+	+	–
Small hands and/or feet	–	–	+	+	+	+	+	+	–	+	+	–	–	+	–	–	–	+	–	+	+	+	+	–
Short fifth finger	+	–	+	+	+	+	–	+	–	+	+	+	+	+	+	+	–	+	+	–	+	+	–	–
Hirsutism	+	–	+	+	+	+	+	–	–	+	+	+	+	+	+	–	+	–	–	+	+	+	+	+
Clinical Score	13	5	11	15	15	17	14	7	4	13	14	8	5	15	9	6	11	6	4	16	17	13	14	6

Individuals with CdLS	25	26	27	28	29	30	31	32	33	34	35	36	37	38	39	40	41	42	43	44	45	46	47	
**Cardinal features (2 points each if present)**																								
Synophrys and/or thick eyebrows	+	+	+	+	+	+	+	+	+	+	+	+	+	+	+	+	+	+	+	+	+	+	+	
Short nose, concave nasal ridge and/or upturned nasal tip	+	+	+	+	–	+	+	–	–	+	–	+	–	+	–	+	+	+	–	+	+	+	+	
Long and/or smooth philtrum	+	+	+	+	–	–	+	+	–	+	+	+	–	+	–	–	–	–	+	+	+	–	+	
Thin upper lip vermilion and/or downturned corners of mouth	+	+	+	+	–	–	–	–	–	+	–	+	–	+	–	+	–	–	+	+	+	+	+	
Hand oligodactyly and/or adactyly	–	–	–	–	–	–	–	–	–	–	–	–	–	+	–	–	–	–	–	–	+	–	–	
Congenital diaphragmatic hernia	–	–	–	–	–	–	–	+	–	–	–	–	–	–	–	–	–	–	–	–	–	–	–	
**Suggestive features (1 point each if present)**																								
Global developmental delay and/or intellectual disability	+	+	+	+	+	+	+	+	+	+	+	+	+	+	+	+	+	+	+	+	+	+	+	
Prenatal growth retardation (< 2 sD)	+	–	–	+	+	–	–	–	+	+	+	+	–	+	+	+	+	+	+	+	+	+	–	
Postnatal growth retardation (< 2 sD)	+	–	+	+	+	–	+	–	+	+	+	+	+	+	–	+	+	+	+	+	+	+	–	
Microcephaly (prenatally and/or postnatally)	+	–	+	+	+	–	+	–	+	+	–	+	–	+	–	+	–	+	+	+	+	+	+	
Small hands and/or feet	+	+	+	+	–	–	–	–	+	+	+	–	+	+	–	+	–	+	–	+	+	+	+	
Short fifth finger	–	–	+	+	+	+	+	+	+	+	+	+	+	–	–	+	–	+	–	+	–	+	+	
Hirsutism	+	+	+	+	+	+	+	–	+	+	–	+	–	+	–	+	–	–	+	+	+	+	+	
Clinical Score	14	11	14	15	8	7	11	8	9	15	9	14	6	16	4	13	7	10	11	15	16	13	13	

Clinical Score: ≥ 11 points, which at least 3 cardinal: classic CdLs; 9–10 points, which at least 2 cardinal: non-classic CdLs; 4–8 points, which at least 1 cardinal: molecular testing; < 4 points: insufficient to indicate molecular testing CdLs. Dotted individuals: involved genes different from *NIPBL*

## Results

**Fig. 1:**
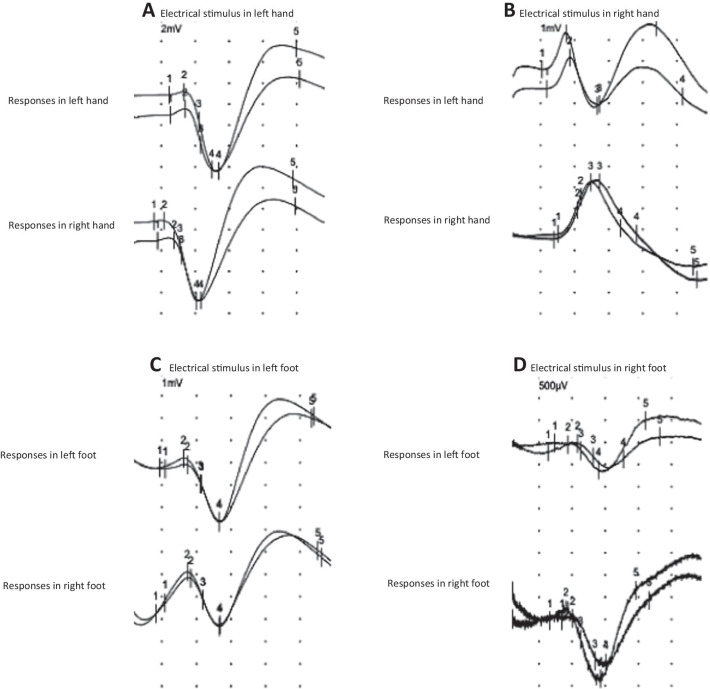
Sympathetic Skin response in upper and lower limbs. **A** Normal symmetrical sympathetic skin response (SSR) in upper limbs in individual -30- after electrical stimulus in left hand, recorded simultaneously in both hands. Upper curves refer to the left hand, lower curves refer to the right hand. **B** Pathological asymmetrical in amplitude and morphology SSR in upper limbs in individual -23- after electrical stimulus in right hand, recorded simultaneously in both hands. Upper curves refer to the left hand, lower curves refer to the right hand. **C** Normal symmetrical normal SSR in lower limbs in individual -30- after electrical stimulus in left foot, recorded simultaneously in both feet. Upper curves refer to the left foot, lower curves refer to the right foot. **D** Pathological symmetrical in amplitude SSR in lower limbs in individual -40- after electrical stimulus in right foot, recorded simultaneously in both feet. Upper curves refer to the left foot, lower curves refer to the right foot

**Fig. 2:**
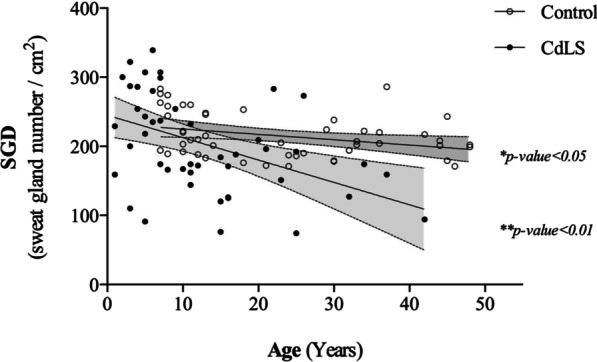
Analysis of SGD. (SGD: sweat gland density: gland number/cm^2^): each dot corresponds to a different individual at the indicated age. Filled dots are CdLS individuals (n = 47) and empty dots correspond to control individuals (n = 50). Lines show mean linear fit and 95% confidence intervals (shadowed areas). Significant non-zero slope, linear regression, *p-value < 0.05, **p-value < 0.01

Conventional motor and sensory nerve conduction studies (large fiber nerves) were normal in all 20 individuals with CdLS analyzed (Additional file 1: Tables 1–3). The study of the autonomic nervous system (small fiber nerves) in HRV at rest was normal as well (Table 2). Nevertheless, SSR revealed mild alterations in lower limbs in 7 of the 20 individuals, with asymmetrical responses (Table 2, Fig. 1). Sudomotor tests evinced reduced SGD in 16 of the 47 individuals with CdLS regarding the control group by decades of life (Table 3). The regression analysis showed that, in spite of dispersion, there were two different populations, with statistically significant differences between the control group and individuals with CdLS (p < 0.05 and p < 0.01) (Fig. 2). The linear regression showed that the slope of the SGD reduction by age is much more pronounced in individuals with CdLS than in controls (Fig. 2). Independence samples T test showed the results of the mean differences of the sweat gland density (SGD) by age group, with reduction in the SGD more evident in the individuals with variants in *NIPBL* than in the controls (p < 0.01). These differences were found in the whole *NIPBL* group as in all the decades of life, except the first one (Fig. 2, Table 4).

**Table 2:**
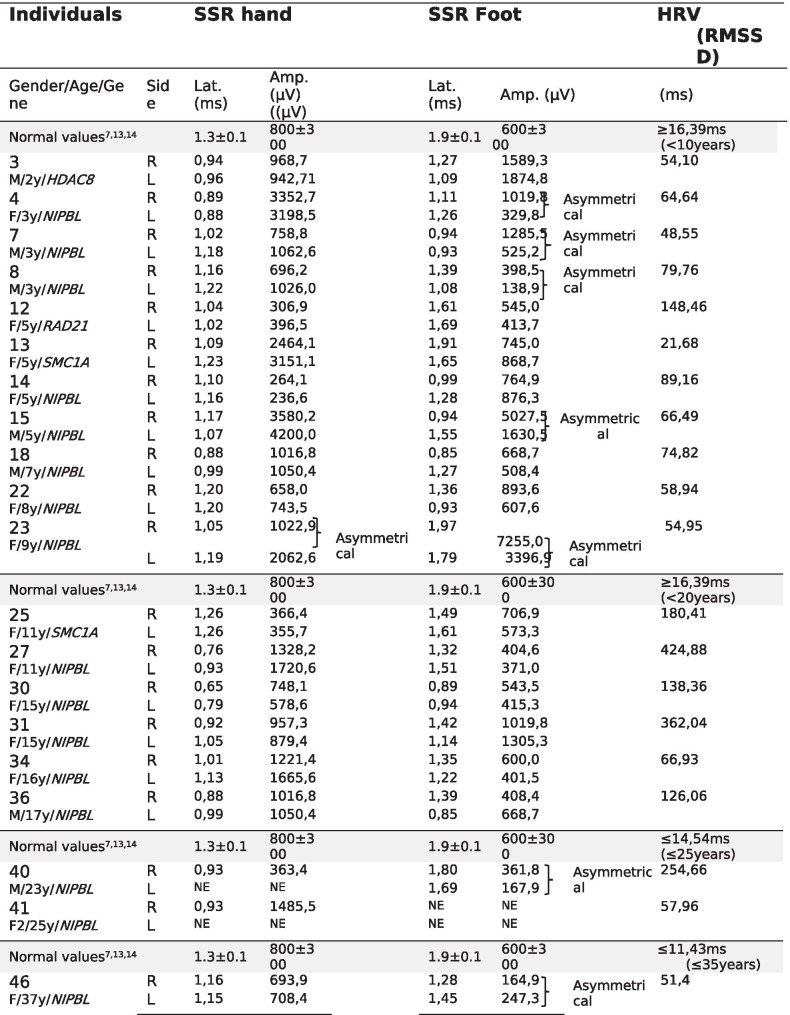
Sympathetic skin response and heart rate variability in CdLS

*SSR* Sympathetic Skin Response; *HRV* Heart Rate Variability; *RMSSD* Root Mean Square of Successive Differences. *Lat* latency; *Amp* Amplitude; *ms* milliseconds; *µV* microvolts; *NE* Not examined. *P40* left arm not studied. *P41* only cooperated for the SSR study in one hand. Asymmetrical: Used to indicate side-to-side differences in amplitude and/or morphology in the Sympathetic Skin Response (SSR) in both upper or lower limbs

**Table 3:**
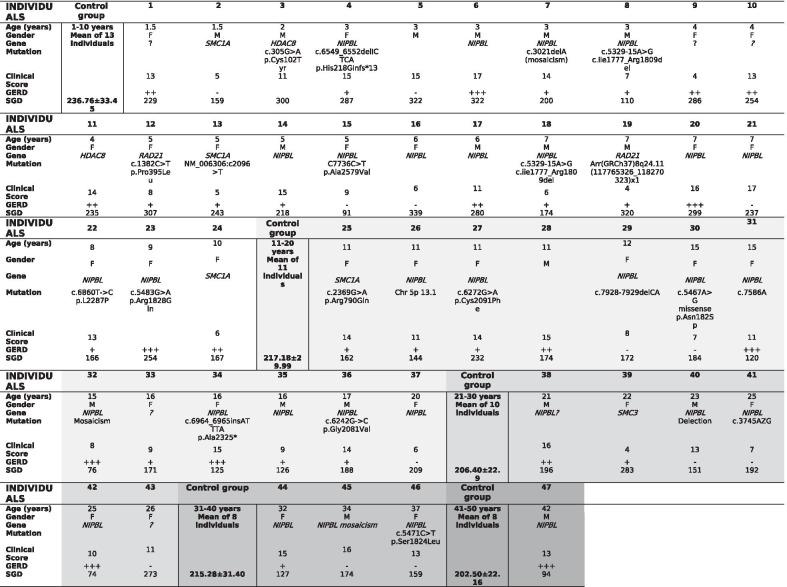
Genetics, clinical score and sweat gland density (SGD) in individuals with CdLS in different decades of life.

Individuals are differentiated in decades of life by different shading colours from: white: 1st decade of life; light grey: 2nd decade of life; medium grey: 3rd decade of life; grey: 4th decade of life; dark grey: 5th decade of life. *M* male, *F* female, *PNS* Peripheral Nervous System, *GERD* Gastroesophageal reflux disease (– no, + mild, +  + moderate, +  +  + severe), *SGD* Sweat Gland Density: gland number/cm^2^

**Table 4:** SGD by decades of life

	Controls	N	CdLS NIPBL	N	p^a^
Mean SGD (g/cm^2^) ± SD	217.60 ± 30.61	50	178.03 ± 72.53	27	0.001
Mean SGD (g/cm^2^) ± SD by decades of life	Controls	N	CdLS NIPBL	N	p^a^
≤ 10 years	236.76 ± 34.81	13	226.00 ± 86.60	10	0.686
11–20 years	217.18 ± 29.99	11	159.09 ± 45.35	11	0.002
21–30 years	206.40 ± 22.9	10	139.00 ± 59.90	3	0.010
31–40 years	215.28 ± 32.40	8	143.00 ± 22.62	2	0.013
41–50 years	202.50 ± 22.16	8	94.0	1	0.002

Values for sweat gland density in CdLS individuals with variants in *NIPBL* and controls by groups of age

^a^Independent samples t test. There are statistically significant differences (p < 0.05) in the SGD global mean (control group compared to the global NIPBL group) and by decades of life, in all the decades except in the first one

Genetic studies of the 47 individuals with CdLS revealed 31 with variants in *NIPBL*, 4 in *SMC1A*, 2 in *RAD21*, 2 in *HDAC8* and 1 in *SMC3* and negative in 7 individuals (Table 3). In Table 3 there are the CdLS Clinical Scores [1]. No relationship between clinical score or gastroesophageal reflux disease (GERD) and findings of the sudomotor test was found. In Additional file 1: Table 4 is shown the SGD in the control group by decades of life.

## Discussion

Though the clinical manifestations of CdLS suggest that the peripheral nervous system is affected, large fiber nerve studies (conventional motor and sensory nerve conduction studies) are within normal limits. However, we have shown evidence, for the first time, for autonomic nervous system dysfunction in individuals with CdLS.

The sympathetic skin response reveals asymmetrical pathological responses in lower limbs in 7 of the 20 individuals (35%), with one of them affected in upper limbs as well. This could be considered a malformative manifestation of the syndrome. However, it is remarkable that the asymmetry is more frequent in lower than in upper limbs, which are often more affected [1–4]. This asymmetry does not seem to be related to GERD or the Clinical Severity Score (CSS), yet all the individuals had mutations in the *NIPBL* gene (Table 2).

Sudomotor testing shows a reduction in the sweat gland density (SGD) in 16 of 47 (34%) of the analyzed individuals with CdLS. These data are further supported by a reduction of the number of sweat droplets imprinted on the silicone after pilocarpine iontophoresis as indirect evidence of decreased postganglionic sudomotor nerve fibers, compared to an unaffected population. Though sweat gland density decreases physiologically with aging, individuals with CdLS show a reduction much greater than should be expected by their age. This decrease is evident from the second decade of life, and is more pronounced at older ages (Table 3, Fig. 2). All of this seems to strengthen the hypothesis that these patients have premature aging. Nevertheless, no relationships were found between SGD reduction and clinical score or GERD.

The reduction in the SGD is evident in individuals with mutations in *NIPBL* (Tables 3, 4), and seems to be similar in individuals with variants in *SMC1A* (3 of the 4 individuals with mutations in *SMC1A* had SGD reduction). However, individuals with variants in *HDAC8* and *RAD 21* are in the first decade of life, so it is early to make an assessment. Surprisingly, there is a high value of sweat gland density in the only individual with an *SMC3* mutation, who is 39 years old. Regarding the ethnic distribution, only 4 individuals in the *NIPBL* group and none in the control group were not Caucasian, and all of them had normal values in SGD, though they were in the first decade of life. In the group of *NIPBL*, there is a repeated mutation, a frameshift mutation in 2 siblings. According to the asymmetry in the SSR response, 3 of the *NIPBL* individuals had missense mutations, 2 of them frameshift mutations and 1 of them splicing mutation, but the number of individuals is not big enough to do a correlation with the autonomic neuropathy. Further studies are warranted to look at autonomic nervous system dysfunction and relation to mutated gene and age in individuals with CdLS.

## Conclusion

Individuals with CdLS have abnormal autonomic nervous system function, showing asymmetries in the sympathetic responses in lower limbs, and pathological results in the sudomotor test. The degree of dysfunction in postganglionic sudomotor nerve fibers might be related to premature aging. Even though, somatic nervous system function studies were normal.

## Supplementary Information

**Additional file 1. Tables 1 to 4:** Motor and Sensory Nerve Conduction Studies Parameters.

## Data Availability

The authors confirm that the data supporting the findings of this study are available within the article and its supplementary materials.

## Abbreviations

CdLS: Cornelia de Lange Syndrome
PNS: Peripheral nervous system
SGD: Sweat gland density
GERD: Gastroesophageal reflux disease
CSS: Clinical severity score
