# Supplementary material for: High rate of autonomic neuropathy in Cornelia de Lange Syndrome
Source: Orphanet J Rare Dis. 2021 Oct 30;16:458. doi: 10.1186/s13023-021-02082-y (PMC8556971; doi:10.1186/s13023-021-02082-y)
Supplement: Supplementary file 1 — Additional file 1. Tables 1 to 4: Motor and Sensory Nerve Conduction Studies Parameters. [file 13023_2021_2082_MOESM1_ESM.docx]

**Supplementary table 1: Sensory Nerve Conduction Studies in CdLS.**

| **SENSORY NERVES** | | **MEDIAN** | | **ULNAR** | | | **RADIAL** | | | **SURAL** | | | **SUPERFICIAL PERONEAL** | | | |
| --- | --- | --- | --- | --- | --- | --- | --- | --- | --- | --- | --- | --- | --- | --- | --- | --- |
| Individual  Gender/age | Side | Amp.  (μV) | Veloc.  (m/s) | | Amp.  (μV) | Veloc.  (m/s) | Amp.  ( μV) | Veloc. (m/s) | Amp.  (μV) | | Veloc.  (m/s) | Amp.  (μV) | | Veloc.  (m/s) |  |  |
| Normal values (2-6y)^11-14^ | | 24.0±7.36 | 46.93±5.03 | | 16.3±2.44 | 44.2±7.79 | 32.01±5.6 | 48.92±32 | 15.41±9.98 | | 49.73±5.53 | 18.29±7.3 | | 48.57±6.1 | | |
| 3  M/2y | R | 70,4 | 50,0 | | 36,5 | 54,5 | 30,0 | 50,0 | 15,1 | | 48,0 | 32,9 | | 56,4 | |  |
|  | L | 57,5 | 54,5 | | 46,1 | 57,1 | 23,5 | 60,0 | 22,8 | | 45,2 | 13,8 | | 51,4 | |  |
| 4  F/3y | R | 40,5 | 52,0 | | 30,7 | 48,1 | 18,4 | 44,2 | 43,4 | | 76,2 | 38,5 | | 46,2 | |  |
|  | L | 80,8 | 52,2 | | 40,2 | 50,0 | 24,5 | 50,0 | 24,5 | | 50,0 | 9,9 | | 37,1 | |  |
| 7  M/3y | R | 31,3 | 51,7 | | 18,0 | 44,0 | 34,5 | 43,5 | 29,0 | | 44,0 | 9,8 | | 48,3 | |  |
|  | L | 20,6 | 55,2 | | NE | NE | NE | NE | 22,1 | | 63,6 | NE | | NE | |  |
| 8  M/3y | R | 64,9 | 54,5 | | 8,3 | 50,0 | 11,9 | 51,6 | 21,3 | | 45,2 | 12,6 | | 48,5 | |  |
|  | L | 20,5 | 51,5 | | 6,6 | 55,6 | 14,3 | 56,0 | 11,4 | | 48,6 | 10,4 | | 42,0 | |  |
| 12  F/5y | R | 37,5 | 53,8 | | 54,2 | 54,5 | 31,6 | 41,0 | 14,5 | | 39,2 | 18,3 | | 43,9 | |  |
|  | L | 64,1 | 55,0 | | 82,2 | 50,0 | 35,3 | 50,0 | 23,0 | | 41,8 | 14,0 | | 40,0 | |  |
| 13  F/5y | R | 45,8 | 56,2 | | 38,4 | 56,0 | NE | NE | 22,0 | | 68,6 | 30,5 | | 53,3 | |  |
|  | L | 43,6 | 58,1 | | 59,6 | 56,0 | NE | NE | 23,7 | | 61,1 | 22,6 | | 53,3 | |  |
| 14  F/5y | R | 63,1 | 54,5 | | 59,3 | 51,6 | 61,3 | 60,9 | 31,8 | | 66,7 | 12,6 | | 63,6 | |  |
|  | L | 72,3 | 61,3 | | 78,3 | 47,1 | 45,4 | 50,0 | 14,7 | | 60,9 | 16,6 | | 57,7 | |  |
| 15  M/5y | R | NE | NE | | NE | NE | NE | NE | NE | | NE | NE | | NE | |  |
|  | L | NE | NE | | NE | NE | NE | NE | NE | | NE | NE | | NE | |  |
| Normal values (7-14y)^11-14^ | | 26.72±9.46 | 53.84±3.26 | | 13.4±4.2 | 46.6±5.6 | 29.89±3.4 | 52.31±6.5 | 26.75±6.79 | | 53.85±4.19 | 28.43±1.5 | | 52.41±5.4 | | |
| 18  M/7y | R | 35,0 | 52,9 | | 12,5 | 56,0 | 13,7 | 54,1 | 16,40 | | 60,0 | 19,1 | | 54,8 | | |
|  | L | 23,6 | 52,8 | | 24,6 | 54,5 | 17,0 | 51,9 | 18,3 | | 57,9 | 20,7 | | 53,1 | | |
| 22  F/8y | R | 42,4 | 61,8 | | 27,7 | 50,0 | 18,2 | 51,7 | 14,0 | | 57,9 | 16,8 | | 59,5 | | |
|  | L | 42,1 | 63,6 | | 38,5 | 66,7 | 33,9 | 50,0 | 8,4 | | 48,5 | 28,0 | | 78,6 | | |
| 23  F/9y | R | 19,0 | 60,6 | | 35,8 | 48,3 | NE | NE | 11,0 | | 47,8 | 27,7 | | 65,2 | | |
|  | L | 36,3 | 67,7 | | 36,5 | 50,0 | NE | NE | 11,4 | | 55,3 | 45,0 | | 48,0 | | |
| 25  F/11y | R | 25,7 | 52,2 | | 12,8 | 50,0 | 30,7 | 56,5 | 26,5 | | 73,3 | 18,8 | | 47,4 | | |
|  | L | 39,1 | 52,0 | | 23,7 | 48,6 | 25,9 | 59,3 | 23,2 | | 49,1 | 15,2 | | 51,2 | | |
| 27  F/11y | R | 35,7 | 55,0 | | 28,5 | 50,4 | NE | NE | 17,3 | | 63,4 | 20,7 | | 74,1 | | |
|  | L | 25,8 | 62,5 | | 17,0 | 55,2 | 12,1 | 80,0 | 14,1 | | 55,0 | 16,8 | | 54,5 | | |
| Normal values (>15y)^11-14^ | | 20.50±3.49 | 53.44±3.19 | | 14.30±2.5 | 53.23±3.5 | 26.76±7.8 | 54.56±6.3 | 18.67±4.39 | | 42.75±4.79 | 20.45±5.2 | | 43.67±4.5 | | |
| 30  F/15y | R | 36,3 | 60,9 | | 32,5 | 55,3 | 19,0 | 60,6 | 21,4 | | 56,4 | 10,4 | | 51,7 | | |
|  | L | 40,0 | 60,9 | | 45,1 | 57,5 | 23,3 | 57,6 | 20,8 | | 55,3 | 11,3 | | 57,9 | | |
| 31  F/15y | R | NE | NE | | NE | NE | NE | NE | 20,8 | | 46,5 | 12,2 | | 51,1 | | |
|  | L | 16,3 | 51,7 | | 73,7 | 37,1 | 38,6 | 52,0 | 13,9 | | 51,3 | 25,4 | | 53,7 | | |
| 34  F/16y | R | 30,0 | 55,0 | | 65,1 | 60,6 | NE | NE | 15,7 | | 41,7 | NE | | NE | | |
|  | L | 46,8 | 55,9 | | 58,0 | 51,9 | 58,0 | 51,9 | 91,3 | | 52,2 | 30,2 | | 51,6 | | |
| 36  M/17y | R | 59,4 | 63,8 | | 41,5 | 45,7 | 27,7 | 56,8 | 16,1 | | NE | 16,1 | | 52,4 | | |
|  | L | 33,5 | 60,0 | | 33,0 | 52,4 | NE | NE | 11,1 | | 56,7 | 13,4 | | 41,7 | | |
| 40  M/23y | R | 36,1 | 66,7 | | 35,9 | 75,9 | 20,9 | 81,8 | 16,2 | | 60,0 | 9,4 | | 58,5 | | |
|  | L | NE | NE | | NE | NE | NE | NE | 20,2 | | 66,7 | 8,4 | | 56,5 | | |
| 41  F/25y | R | 51,0 | 71,8 | | NE | NE | NE | NE | NE | | NE | NE | | NE | | |
|  | L | 42,7 | NE | | NE | NE | NE | NE | NE | | NE | NE | | NE | | |
| 46  F/37y | R | 57,7 | 65,0 | | 40,1 | 60,6 | NE | NE | 17,6 | | 53,6 | 15,4 | | 58,3 | | |
|  | L | 58,9 | 65,0 | | 37,7 | 66,7 | NE | NE | 30,0 | | 68,3 | 16,5 | | 63,4 | | |

Lat: latency, Amp: Amplitude, Veloc: Velocity, µs: microseconds, mV: millivolts, m/s: meters per second. NE: Not examined. P15 did not cooperate for the nerve conduction study. P40 nerves of left arm not studied. P41 only cooperated for the study of both median nerves.

**Supplementary table 2: Motor Nerve Conduction Studies in upper limbs in CdLS**.

| **MOTOR NERVES** | | **MEDIAN** | | | **ULNAR** | | | | | |
| --- | --- | --- | --- | --- | --- | --- | --- | --- | --- | --- |
| Individual  Gender/age | Side | Lat.  (ms) | Amp.  (mV) | Veloc.  (m/s) | Lat.  (ms) | Amp.  (mV) | Veloc.  (m/s) | F lat.  (ms) | F % |  |
| Normal values (1-3y)^11-14^ | | 2.18±0.43 | 9.55±4.34 | 53.59±5.29 | 1.86±0.16 | 8.90±3.61 | 52.4±5.23 | 14.07±0.4 |  |  |
| 3  M/2y | R | 2,00 | 6,5 | 62,2 | 1,65 | 7,8 | 66,7 | 14,50 | 75 |  |
|  | L | 2,15 | 7,0 | 52,6 | 1,6 | 7,4 | 60,0 | 14,85 | 40 |  |
| 4  F/3y | R | 1,75 | 6,0 | 61,3 | 1,80 | 10,6 | 71,4 | 13,20 | 50 |  |
|  | L | 2,05 | 6,8 | 57,9 | 2,00 | 10,1 | 56,5 | 13,25 | 66 |  |
| 7  M/3y | R | 1,60 | 2,3 | 43,9 | 1,15 | 8,0 | 63,0 | 14,25 | 50 |  |
|  | L | 2,10 | 3,1 | 73,1 | NE | NE | NE | NE | NE |  |
| 8  M/3y | R | 1,90 | 7,7 | 53,8 | 1,60 | 7,3 | 72,2 | 14,85 | 100 |  |
|  | L | 2,20 | 5,7 | 57,8 | 1,25 | 9,0 | 66,7 | 14,25 | 20 |  |
| Normal values (4-6y)^11-14^ | | 2.27±0.45 | 10.37±3.66 | 56.26±4.61 | 2.03±0.54 | 9.24±2.54 | 56.1±4.97 | 15.9±0.8 |  |  |
| 12  F5/y | R | 2,75 | 8,8 | 59,4 | 2,20 | 7,9 | 63,6 | 18,05 | 75 |  |
|  | L | 2,85 | 9,1 | 51,4 | 2,25 | 8,7 | 67,5 | 18,30 | 100 |  |
| 13  F/5y | R | 2,20 | 8,5 | 56,0 | 1,40 | 8,9 | 70,3 | 13,90 | 100 |  |
|  | L | 2,45 | 9,0 | 61,2 | 1,50 | 7,6 | 65,1 | 15,30 | 20 |  |
| 14  F/5y | R | 2,35 | 9,2 | 52,8 | 2,00 | 8,5 | 60,7 | 17,10 | 100 |  |
|  | L | 2,10 | 9,1 | 64,2 | 2,20 | 10,2 | 60,0 | 16,50 | 66,7 |  |
| 15  M/5y | R | NE | NE | NE | NE | NE | NE | NE | NE |  |
|  | L | NE | NE | NE | NE | NE | NE | NE | NE |  |
| Normal values (7-14y)^11-14^ | | 2.73±0.44 | 11.25±0.45 | 57.32±3.35 | 2.25±0.13 | 13.45±4.3 | 58.2±9.7 | 19.1±1.0 |  |  |
| 18  M/7y | R | 2,65 | 7,8 | 54,8 | 1,60 | 12,1 | 61,1 | 17,30 | 57,1 |  |
|  | L | 2,60 | 7,8 | 54,2 | 1,45 | 10,2 | 57,1 | 16,35 | 50 |  |
| 22  F/8y | R | 2,15 | 8,6 | 78,9 | 1,85 | 9,3 | 64,9 | 15,50 | 42,9 |  |
|  | L | 2,35 | 10,9 | 73,2 | 1,30 | 8,5 | 62,5 | 15,55 | 20 |  |
| 23  F/9y | R | 2,25 | 7,6 | 66,7 | 1,50 | 7,0 | 57,1 | 14,80 | 33,3 |  |
|  | L | 2,15 | 5,6 | 78,8 | 1,50 | 5,5 | 81,8 | 14,10 | 42,90 |  |
| 25  F/11y | R | 4,05 | 8,7 | 45,9 | 2,40 | 10,2 | 38,7 | 24,75 | 57,1 |  |
|  | L | 2,45 | 14,2 | 56,2 | 2,30 | 11,2 | 52,2 | 25,55 | 80 |  |
| 27  F/11y | R | 2,35 | 6,7 | 59,5 | 2,55 | 1,3 | NE | NE | NE |  |
|  | L | 2,25 | 8,3 | 59,5 | 1,25 | 12,8 | 58,8 | 15,55 | 100 |  |
| Normal values (>14y)^11-14^ | | 2.89±0.67 | 10.55±5.24 | 10.55±5.24 | 54.34±5.34 | 2.54±0.76 | 12.87±2.76 | 20.4±8.4 |  |  |
| 30  F/15y | R | 2,95 | 10,7 | 61,5 | 1,90 | 9,2 | 62,7 | 23,45 | 40 |  |
|  | L | 2,85 | 9,5 | 59,7 | 1,90 | 8,7 | 61,2 | 22,45 | 45,5 |  |
| 31  F/15y | R | 2,05 | 5,6 | 56,7 | 2,30 | 3,8 | NE | 14,15 | 28,60 |  |
|  | L | 2,15 | 8,0 | 68,3 | 2,55 | 5,7 | 71,4 | 16,05 | 50 |  |
| 34  F/16y | R | 2,35 | 3,4 | 51,0 | 1,80 | 9,7 | 66,7 | 17,75 | 60 |  |
|  | L | 2,95 | 7,5 | 80,0 | 2,90 | 8,9 | 52,9 | 18,40 | 66,7 |  |
| 36  M/17y | R | 3,05 | 13,9 | 52,6 | 2,25 | 8,4 | 52,5 | 27,45 | 100 |  |
|  | L | 3,55 | 15,5 | 59,0 | 2,20 | 7,8 | 52,8 | 26,85 | 57,1 |  |
| 40  M/23y | R | 2,55 | 13,8 | 64,5 | 1,80 | 10,0 | 75,4 | 19,70 | 25 |  |
|  | L | NE | NE | NE | NE | NE | NE | NE | NE |  |
| 41  F/25y | R | 2,70 | 8,7 | NE | NE | NE | NE | NE | NE |  |
|  | L | 2,75 | 8,6 | NE | NE | NE | NE | NE | NE |  |
| 46  F/37y | R | 2,65 | 6,4 | 63,2 | 1,90 | 11,7 | 66,7 | 18,00 | 100 |  |
|  | L | 2,30 | 9,1 | 64,2 | 1,90 | 12,6 | 60,0 | 19,30 | 100 |  |

Lat: latency, Amp: Amplitude, Veloc: Velocity, ms: milliseconds, m/s: meters per second, F: F wave response. H: H reflex, Am: amplitude. NE: Not examined. P15 did not cooperate for the nerve conduction study. P40 nerves of left arm not studied. P41 only cooperated for the study of both median nerves.

**Supplementary table 3:** **Motor Nerve Conduction Studies in lower limbs in CdLS.**

| **MOTOR NERVES** | | **PERONEAL** | | | | | **POSTERIOR TIBIAL** | | | | | | | | | | | |
| --- | --- | --- | --- | --- | --- | --- | --- | --- | --- | --- | --- | --- | --- | --- | --- | --- | --- | --- |
| Individual  Gender/Age | Side | Lat.  (ms) | Amp.  (mV) | | Veloc.  (m/s) | Lat.  (ms) | | Amp.  (mV) | | VeLoc.  (m/s) | | F lat.  (m/s) | F % | | H Lat.  (m/s) | | H Amp.  (mV) | |
| Normal values (1-3y)^11-15^ | | 2.62±0.75 | | 6.10±2.99 | 55.73±4.45 | 2.45±0.4 | | | 9.07±2.12 | | 42.6±3.80 | 15.71±1.79 | |  | 18.7±7.2 | 2.4 ±1.4 | |  |
| 3 | R | 2,55 | 3,4 | | 47,8 | 1,85 | | 7,5 | | 50,9 | | 20,15 | 50 | | 13,70 | | 1,4 | |
| M/2y | L | 1,45 | 2,7 | | 54,5 | 2,10 | | 7,1 | | 51,7 | | 19,95 | 100 | | 13,65 | | 1,1 | |
| 4 | R | 3,20 | 3,1 | | 44,9 | 2,25 | | 14,6 | | 43,3 | | 19,50 | 100 | | 15,80 | | 1,0 | |
| F/3y | L | 2,85 | 4,6 | | 50,0 | 2,15 | | 12,4 | | 44,8 | | 20,05 | 100 | | 16,00 | | 0,4 | |
| 7 | R | NE | NE | | NE | 1,90 | | 9,9 | | 65,2 | | 19,80 | 100 | | NE | | NE | |
| M/3y | L | NE | NE | | NE | 2,05 | | 10,1 | | 53,2 | | 21,00 | 100 | | NE | | NE | |
| 8 | R | 2,30 | 7,4 | | 44,1 | 2,45 | | 22,4 | | 52,1 | | 24,15 | 75 | | 17,90 | | 1,1 | |
| M/3y | L | 2,75 | 4,9 | | 56,5 | 2,25 | | 18,1 | | 54,8 | | 22,90 | 100 | | 15,15 | | 1,3 | |
| Normal values (4-6y)^11-15^ | | 3.01±0.43 | 7.10±4.76 | | 56.14±4.96 | 2.85±0.2 | | 10.12±5.3 | | 43.76±2.3 | | 15.75±1.77 |  | | 18.7±7.2 | | 2.4 ±1.4 | |
| 12 | R | 3,55 | 3,4 | | 58,2 | 3,15 | | 9,5 | | 41,0 | | 33,90 | 100 | | 22,20 | | 0,3 | |
| F/5y | L | 4,65 | 2,2 | | 55,6 | 3,20 | | 7,9 | | 41,5 | | 32,35 | 100 | | 22,65 | | 0,2 | |
| 13 | R | 1,50 | 6,4 | | 50,7 | 2,10 | | 12,1 | | 52,5 | | 21,25 | 100 | | 17,60 | | 0,4 | |
| F/5y | L | 2,00 | 4,7 | | 63,2 | 2,15 | | 13,7 | | 50,6 | | 20,55 | 60 | | 16,55 | | 1,5 | |
| 14 | R | 2,95 | 4,9 | | 51,2 | 1,35 | | 13,1 | | 42,1 | | 28,00 | 100 | | 19,65 | | 1,1 | |
| F/5y | L | 3,10 | 6,7 | | 52,8 | 2,35 | | 11,8 | | 53,6 | | 28,00 | 100 | | 19,60 | | 0,6 | |
| 15 | R | NE | NE | | NE | NE | | NE | | NE | | NE | NE | | NE | | NE | |
| M/5y | L | NE | NE | | NE | NE | | NE | | NE | | NE | NE | | NE | | NE | |
| Normal values (7-14y)^11-15^ | | 3.25±0.51 | 8.15±4.19 | | 57.05±4.54 | 3.13±0.2 | | 12.25±6.3 | | 45.67±1.23 | | 19.89±7,4 |  | | 18.7±7.2 | | 2.4 ±1.4 | |
| 18 | R | 3,25 | 5,7 | | 58,1 | 2,45 | | 18,6 | | 52,4 | | 29,45 | 100 | | 23,10 | | 1,5 | |
| M/7y | L | 3,25 | 6,3 | | 51,9 | 2,70 | | 18,4 | | 50,9 | | 29,95 | 75 | | 24,10 | | 0,9 | |
| 22 | R | 2,70 | 5,8 | | 58,9 | 2,55 | | 17,7 | | 62,7 | | 24,90 | 100 | | 18,70 | | 1,4 | |
| F/8y | L | 2,80 | 6,0 | | 59,3 | 3,85 | | 11,1 | | 68,5 | | 29,75 | 100 | | 19,25 | | 1,4 | |
| 23 | R | 4,10 | 2,4 | | 59,3 | 2,25 | | 5,9 | | 54,5 | | 28,55 | 100 | | NE | | NE | |
| F/9y | L | 2,95 | 4,5 | | 54,3 | 2,80 | | 11,8 | | 53,2 | | 30,25 | 100 | | NE | | NE | |
| 25 | R | 3,95 | 4,7 | | 45,2 | 2,95 | | 13,0 | | 43,4 | | 37,40 | 100 | | 25,90 | | 1,5 | |
| F/11y | L | 3,75 | 4,4 | | 40,4 | 2,70 | | 12,5 | | 48,0 | | 36,70 | 100 | | 25,35 | | 1,2 | |
| 27 | R | 2,60 | 8,1 | | 62,5 | 1,85 | | 13,2 | | 67,4 | | 27,65 | 100 | | 17,30 | | 0,2 | |
| F/11y | L | 2,10 | 9,3 | | 57,8 | 2,25 | | 7,9 | | 60,8 | | 27,40 | 100 | | 20,95 | | 0,4 | |
| Normal values (>14y)^11-15^ | | 3.65±0.87 | 7.65±6.54 | | 56.34±2.3 | 3.45±2.45 | | 10.56±2.3 | | 45.65±7.6 | | 29.79±8,7 |  | | 18.7±7.2 | | 2.4 ±1.4 | |
| 30 | R | 3,60 | 8,2 | | 57,6 | 2,85 | | 14,2 | | 57,1 | | 41,50 | 80 | | 27,40 | | 1,2 | |
| F/15y | L | 4,30 | 6,1 | | 54,5 | 3,00 | | 13,8 | | 54,2 | | 41,50 | 100 | | 27,80 | | 2,3 | |
| 31 | R | 3,80 | 8,9 | | 53,5 | 3,85 | | 7,6 | | 74,4 | | 31,60 | 100 | | 20,90 | | 1,5 | |
| F/15y | L | 3,85 | 6,0 | | 61,1 | 3,20 | | 6,4 | | 43,6 | | 30,90 | 62,5 | | 20,50 | | 1,3 | |
| 34 | R | NE | NE | | NE | 3,45 | | NE | | NE | | 30,20 | 33,3 | | 20,50 | | 0,4 | |
| F/16y | L | 3,80 | 5,2 | | 44,3 | 7,8 | | 48,1 | | NE | | 33,55 | 75 | | 25,25 | | 0,6 | |
| 36 | R | 4,55 | 3,9 | | 52,0 | 4,00 | | 10,6 | | 49,4 | | 47,05 | 100 | | 29,00 | | 2,7 | |
| M/17y | L | 4,35 | 6,1 | | 52,0 | 5,00 | | 12,7 | | 48,0 | | 48,05 | 80 | | 28,90 | | 2,8 | |
| 40 | R | 3,30 | 4,0 | | 53,8 | 3,35 | | 10,7 | | 47,6 | | 40,70 | 63,6 | | 25,9 | | 3,3 | |
| M/23y | L | 3,40 | 3,4 | | 64,5 | 3,05 | | 10,8 | | 52,2 | | 40,15 | 71,4 | | 26,10 | | 1,6 | |
| 41 | R | NE | NE | | NE | NE | | NE | | NE | | NE | NE | | NE | | NE | |
| F/25y | L | NE | NE | | NE | NE | | NE | | NE | | NE | NE | | NE | | NE | |
| 46 | R | 2,75 | 4,3 | | 59,3 | 2,60 | | 11,3 | | 56,9 | | 35,90 | 100 | | 24,35 | | 3,7 | |
| F/37y | L | 3,10 | 11,0 | | 59,8 | 3,10 | | 14,2 | | 57,6 | | 34,25 | 100 | | 25,00 | | 0,6 | |

Lat: latency, Amp: Amplitude, Veloc: Velocity, ms: milliseconds, m/s: meters per second, F: F wave response. H: H reflex, Am: amplitude. NE: Not examined. P15 did not cooperate for the nerve conduction study.

| **Mean control group** | **Controls** | **C1** | **C2** | **C3** | **C4** | **C5** | **C6** | **C7** | **C8** | **C9** | **C10** | **C11** | **C12** | **C13** |
| --- | --- | --- | --- | --- | --- | --- | --- | --- | --- | --- | --- | --- | --- | --- |
| **1-10 years** | **Age** | 7 | 7 | 7 | 7 | 8 | 8 | 8 | 8 | 10 | 10 | 10 | 10 | 10 |
|  | **Gender** | F | M | M | M | F | M | M | F | M | M | M | F | M |
| **236.76±34.81** | **SGD (g/cm^2^)** | 276 | 194 | 263 | 283 | 274 | 244 | 189 | 258 | 222 | 203 | 192 | 220 | 260 |
| **Mean control group** | **Controls** | **C14** | **C15** | **C16** | **C17** | **C18** | **C19** | **C20** | **C21** | **C22** | **C23** | **C24** |  |  |
| **11-20 years** | **Age** | 11 | 11 | 12 | 12 | 13 | 13 | 13 | 13 | 14 | 18 | 18 |  |  |
|  | **Gender** | M | F | F | F | F | M | F | F | M | F | F |  |  |
| **217.18±29.99** | **SGD (g/cm^2^)** | 260 | 209 | 210 | 188 | 215 | 246 | 248 | 183 | 201 | 253 | 176 |  |  |
| **Mean control group** | **Controls** | **C25** | **C26** | **C27** | **C28** | **C29** | **C30** | **C31** | **C32** | **C33** | **C34** |  |  |  |
| **21-30 years** | **Age** | 21 | 23 | 24 | 24 | 25 | 26 | 29 | 30 | 30 | 30 |  |  |  |
|  | **Gender** | M | F | M | M | F | F | F | F | F | F |  |  |  |
| **206.40±22.9** | **SGD (g/cm^2^)** | 171 | 205 | 171 | 187 | 186 | 190 | 224 | 179 | 178 | 238 |  |  |  |
| **Mean control group** | **Controls** | **C35** | **C36** | **C37** | **C38** | **C39** | **C40** | **C41** | **C42** |  |  |  |  |  |
| **31-40 years** | **Age** | 32 | 32 | 33 | 33 | 34 | 36 | 36 | 37 |  |  |  |  |  |
|  | **Gender** | F | F | F | F | M | F | F | M |  |  |  |  |  |
| **215.28±32.40** | **SGD (g/cm^2^)** | 194 | 194 | 202 | 207 | 222 | 220 | 204 | 286 |  |  |  |  |  |
| **Mean control group** | **Controls** | **C43** | **C44** | **C45** | **C46** | **C47** | **C48** | **C49** | **C50** |  |  |  |  |  |
| **41-50 years** | **Age** | 42 | 44 | 44 | 45 | 45 | 46 | 48 | 48 |  |  |  |  |  |
|  | **Gender** | F | F | F | F | F | F | M | F |  |  |  |  |  |
| **202.50±22.16** | **SGD (g/cm^2^)** | 217 | 201 | 208 | 243 | 179 | 171 | 202 | 199 |  |  |  |  |  |

**Supplementary table 4: Control Group Sweat Gland Density values**

SGD= Sweat gland density: sweat gland number/cm^2^. Controls are differentiated in decades of life by different shading colors from: white: 1^st^ decade of life; light grey: 2^nd^ decade of life; medium grey: 3^rd^ decade of life; grey: 4^th^ decade of life; dark grey: 5^th^ decade of life. M=male, F=female.
